# Does Oxidative Stress Induced by Alcohol Consumption Affect Orthodontic Treatment Outcome?

**DOI:** 10.3389/fphys.2017.00022

**Published:** 2017-01-25

**Authors:** Jorge M. Barcia, Sandra Portolés, Laura Portolés, Alba C. Urdaneta, Verónica Ausina, Gema M. A. Pérez-Pastor, Francisco J. Romero, Vincent M. Villar

**Affiliations:** ^1^School of Medicine and Dentistry, Universidad Católica de Valencia San Vicente MártirValencia, Spain; ^2^Facultad de Ciencias de la Salud, Universidad Europea de ValenciaValencia, Spain; ^3^Department of Biomedical Sciences, Universidad Cardenal Herrera, CEUMoncada, Spain

**Keywords:** ethanol, oxidative stress, orthodontic movement, periodontal ligament, orthodontic treatment

## Abstract

**HIGHLIGHTS**

- Ethanol, Periodontal ligament, Extracellular matrix, Orthodontic movement.

Alcohol is a legal drug present in several drinks commonly used worldwide (chemically known as ethyl alcohol or ethanol). Alcohol consumption is associated with several disease conditions, ranging from mental disorders to organic alterations. One of the most deleterious effects of ethanol metabolism is related to oxidative stress. This promotes cellular alterations associated with inflammatory processes that eventually lead to cell death or cell cycle arrest, among others. Alcohol intake leads to bone destruction and modifies the expression of interleukins, metalloproteinases and other pro-inflammatory signals involving GSKβ, Rho, and ERK pathways. Orthodontic treatment implicates mechanical forces on teeth. Interestingly, the extra- and intra-cellular responses of periodontal cells to mechanical movement show a suggestive similarity with the effects induced by ethanol metabolism on bone and other cell types. Several clinical traits such as age, presence of systemic diseases or pharmacological treatments, are taken into account when planning orthodontic treatments. However, little is known about the potential role of the oxidative conditions induced by ethanol intake as a possible setback for orthodontic treatment in adults.

## Introduction

Since prehistoric ages alcohol (chemically known as ethyl alcohol or ethanol; EtOH) has been consumed by humans. In fact, EtOH is present in beer, wine, spirits and many other drinks. In fact, these products are usually consumed on a daily basis in several countries worldwide. EtOH dependence and abuse are the most abundant mental disorders worldwide. In America, approximately 14% of the population meets chronic alcoholic criteria during some period of their lives (Elkstrom and Ingelman-Sundberg, 1989; Caro and Cederbaum, 2004). EtOH is involved in almost 50% of traffic accidents, the majority of homicides, suicides and domestic violence cases (Graham et al., 1998; Ofori-Adjei et al., 2007). Additionally, EtOH is also implicated in several organic diseases as well as in diverse forms of cancer, including oral cancer (Nelson et al., 2013).

Many reports are available on EtOH-related oral health (Kranzler et al., 1990; Franceschi, 1993; Gelbier and Harris, 1996). There are some important studies on gingival margin recession and oral mucosae alterations (Harris et al., 1996, 2004; Khocht et al., 2003, 2013). Little evidence is reported about the direct effects of EtOH consumption on dental tissues, aside from cancer. One important reason to explain this lack of evidence is the existence of a diversity of factors in connection with alcoholism, e.g., vitamin/nutrients deficiency, other drugs of abuse, smoking, deficient oral care, caries, etc. All these elements impair the identification of concrete factors directly and exclusively related to EtOH in oral diseases (Gelbier and Harris, 1996; Marques et al., 2015).

According to the American Association of Orthodontists, around 4.8 million Americans wear braces. From 1994 to 2010, the percentage of adults getting braces rose from 680,000 to 1.1 million a year (58%). This fact suggests that a relevant number of adults getting braces or other tooth-related devices might consume EtOH along a great part of the orthodontic treatment.

There are several, and sometimes unknown, factors that determine orthodontic outcome. This review focuses on the potential role of EtOH consumption during orthodontic treatment as a plausible factor affecting orthodontic outcome. Surprisingly, EtOH exposure and orthodontic movement affect the same cellular and molecular signaling pathways, giving support to this hypothesis.

## General and local EtOH metabolism: oxidative stress and EtOH-related diseases

Because the liver is the main EtOH-detoxifying organ, EtOH-induced alterations have been mostly studied in hepatic tissue. However, nervous tissue, diverse connective-related tissues and others are also affected. EtOH exerts its deleterious effects in several tissues via oxidative and non-oxidative metabolic pathways (Bondy and Guo, 1995) involving free radical production and lipid peroxidation (Sun et al., 1997; Bosch-Morell et al., 1998; Ramachandran et al., 2003; Almansa et al., 2013; Flores-Bellver et al., 2014). One of the most important factor in this toxic process deals with the properties of EtOH to promote reactive oxygen species (ROS). These ROS ultimately react with macromolecules, among them membrane lipids, producing aldehydes such as 4-Hydroxynonenal (4-HNE) and Malondialdehyde (MDA). It is well known that aldehydes and ROS can directly affect both proteins or DNA, leading to transcription-repression of concrete genes. In fact, the role of ROS and aldehydes seems to be a key factor for these alterations, partially confirmed by the fact that administration of antioxidants prevents these EtOH-induced cellular alterations (Herrera et al., 2003; Bati et al., 2015; Han et al., 2015).

Cytochrome P450 and alcohol dehydrogenase (ADH) are the most relevant enzymes involved in EtOH metabolism. Both enzymes can be found not only in liver but also in other tissues (detailed below). The cytochrome P450 family proteins are involved in the oxidative metabolism of both endogenous and xenobiotic products (Tsutsumi et al., 1993; Miksys and Tyndale, 2002). It is known that CYP2E1 isoform is specifically involved in EtOH oxidation; furthermore, CYP2E1 has more affinity for EtOH than alcohol dehydrogenase (ADH) (Albano, 2008). In fact, CYP2E1 assumes an important role in ethanol metabolism, being considered as a major component of the microsomal ethanol-oxidizing system (MEOS) (Lieber and DeCarli, 1970; Koop et al., 1982). Despite EtOH being mostly catabolized in the liver by CYP2E1, the presence of CYP2E1 and ADH in other tissues indicates that EtOH could also be processed by a non-hepatic route (Martinez-Gil et al., 2015).

CYP2E1 is present in the digestive system, one of the most threatening environments because it is continuously exposed to different media containing chemicals, toxins, etc. In fact, CYP2E1 and ADH are strongly expressed not only in liver and the digestive tract, but also in other human oral cells as gingival fibroblasts, pulp, tongue and osteoblasts (Redetzki, 1960; Dong et al., 1996; Chen et al., 2006; Reichl et al., 2010; Plapp et al., 2015). Interestingly enough, it is well established that there is a good relation between CYP2E1 and EtOH in several digestive-related forms of cancer, e.g., mouth, pharynx, esophagus, colorectum and liver cancer (reviewed by Seitz and Wang, 2013). The presence of CYP2E1 and ADH in other cell types could explain a local and direct EtOH-detoxifying process (Flores-Bellver et al., 2014). In this sense, ethanol diffuses rapidly into saliva. Thirty minutes after alcohol intake, EtOH salivary and plasmatic levels are equilibrated. At the same time the levels of acetaldehyde in saliva exceed the systemic blood levels. Acetaldehyde and ethanol from saliva easily reach all the local tissues (Waszkiewicz et al., 2011, 2012; Zalewska et al., 2011). So it seems reasonable that EtOH and acetaldehyde can directly affect oral related structures.

Despite the fact that ADH has lower affinity for EtOH than CYP2E1, ADH is also relevant for EtOH detoxification. CYP2E1 and ADH are both present in the liver (Redetzki, 1960; Plapp et al., 2015) and also expressed in human attached gingiva and tongue (Dong et al., 1996). Surprisingly enough, whereas ADH is expressed in stromal osteoblasts, CYP2E1 seems to be unexpressed (Chen et al., 2006). Although these enzymes are not ubiquitously present in all tissues, their presence in liver and other tissues, clearly indicates the existence of extra-hepatic EtOH metabolism and that it might be related with some EtOH-related forms of cancer (Seitz and Wang, 2013).

## Periodontum, extracellular matrix, and bone dynamics

The periodontum must be briefly presented as a complex histological area surrounding teeth relevant for root-tooth stability. This periodontal structure includes fibroblasts surrounded by the extracellular matrix (ECM) of hyaluronic acid (HA) and other extracellular proteins as collagen, mostly produced by periodontal fibroblasts. The most abundant collagen form is the type I collagen (Bornstein and Sage, 1980; Zhang et al., 1993).

Other important components of the periodontal ligament (PDL) are the matrix metalloproteinase enzyme family (MMP's) that degrade collagen, and its counterpart, tissue inhibitor metalloproteinases (TIMP) that do inhibit MMP's, being MMP-1 enzyme the most abundant in PDL (Birkedal-Hansen et al., 1993). Obviously, the balance between collagen production and MMP's activity determines the PDL quality and consequently dental stability. MMP's also degrade collagen under pathological conditions and therefore MMP-1, MMP-8, MMP-2, MMP-13 are locally and temporarily expressed during tooth movement phases (Apajalahti et al., 2003; Ingman et al., 2005; Cantarella et al., 2006; Leonardi et al., 2006; Huang et al., 2008; Meeran, 2012).

Root and bone resorption are both directly regulated by a group of tumor necrosis factor (TNF)-related proteins with paracrine-regulatory properties (Schoppet et al., 2002). Osteoprotegerin (OPG) is a soluble protein secreted by osteoblasts that acts as an inhibitor of both osteoclast differentiation and resorptive activity, promoting osteoclast apoptosis (Oshiro et al., 2002). Receptor activator of nuclear factor kappa-b ligand (RANKL) is expressed on the cell surface of osteoblast precursors (Schoppet et al., 2002), whereas its receptor (RANK) is expressed by osteoblastic cell lineages and activated T-cells (Katagiri and Takahashi, 2002). RANKL acts, together with macrophage colony stimulating-factor (M-CSF), promoting osteoclast formation, differentiation and activation, enhancing bone resorption activities (Kong et al., 1999; Liu and Zhang, 2015; Martin and Sims, 2015).

One important step for osteoclast fusion and activation is the coupling of RANK to RANKL. This union can be blocked by OPG, so the balance “resorption vs. reposition” depends on the prevalence of RANK vs. OPG, respectively.

## Orthodontic forces affect periodontal structures modifying intra- and extra-cellular proteins

### Orthodontic forces lead to extracellular modifications

During orthodontic movement, applied forces modulate both molecular and cellular configurations, e.g., those producing collagen (Bumann et al., 1997), modifying the periodontal structure and therefore dental position (Nakagawa et al., 1994; Krishnan and Davidovitch, 2006). Some evidence indicates that mechanical forces modulate the expression of integrins, MMP's or collagen (Bolcato-Bellemin et al., 2000; Von den Hoff, 2003; He et al., 2004). On the hypothetical model for periodontal remodeling summarized by Meikle (2006), tension and compressive sides present some similarities. In the tensile strain, periodontal fibroblasts release IL-1 and IL-6; these interleukins can stimulate MMP's and inhibit TIMP synthesis, so bone and matrix lose structure in order to facilitate bone and PDL regeneration. At the same time, mechanically activated fibroblasts can induce angiogenesis by vascular endothelial growth factor (VEGF) release, helping bone renewal. In the compression side, similarly to the tensile side, IL-1, IL-6, and MMP's are released. One of the differences between both complementary processes seems to be the prevalence of OPG vs. RANK, leading to bone reconstruction and bone destruction, respectively (Tyrovola et al., 2008).

This represents an interesting issue for orthodontics or periodontal management, since both conditions involve these type of cellular responses, e.g., during tooth movement or periodontal disease. In this regard, some reports have found different biological markers in the gingivo-crevicular fluid (GCF): elevated levels of Prostaglandin E, IL-1β, IL-6, TNF-α and epidermal growth factor (EGF) have been found in GCF during tooth movement or periodontal disease (Grieve et al., 1994; Uematsu et al., 1996). Hyaline material and sterile necrosis in local pressure zones have been found also after tooth movement (Kurol and Owman-Moll, 1998). Unfortunately, the significance of these changes is only partially known.

Extracellular matrix degradation facilitates cell proliferation and capillary growth leading to the synthesis of new PDL and bone structures. However, on the compression side, periodontal cells also release IL-1 and IL-6, up-regulating not only MMP's, but also RANKL, leading to osteoclast-mediated bone resorption (Nakano et al., 2011).

Cathepsins are lysosomal cysteine proteases that play an important role in bone resorption. Cathepsin B levels can be increased by orthodontic tooth movement, being involved in extracellular matrix degradation in response to mechanical stress (Maeda et al., 2007). Since Cathepsins K, B and L are over-expressed in the compression side, they may be related to bone resorption (Domon et al., 1999; Sugiyama et al., 2003).

### Orthodontic forces lead to intracellular modifications

Mechanical strain generates diverse intracellular responses in cells during orthodontic movements that could be of clinical interest. Integrins are transmembrane proteins whose extracellular side connects to the ECM via fibronectin (Wang et al., 1993; Clarke and Brugge, 1995), and the intracellular one connects with actin of the cytoskeleton. In fact, this actin-cytoskeletal connection is mediated by proteins as paxillin, talin and vinculin leading to focal adhesions that are crucial for cell adhesion and migration (Sastry and Burridge, 2000; Meikle, 2006). Although little is known about the role of integrin receptors in ECM for cell adhesion, the intracellular side is associated to cAMP and inositol phosphate activation pathways both involved in downstream cell signaling (Wang et al., 1993; DeMali et al., 2003). Well known integrin-mediated extracellular signals are mitogen-activated protein kinases (MAPKs) and Rho pathways, both are activated by mechanic stimuli in periodontal fibroblasts and osteoblasts (Basdra et al., 1995; Peverali et al., 2001).

MAPKs regulate several cellular responses such as cell division, metabolic processes, survival-apoptosis and differentiation, among others. Five distinct groups of MAPKs have been characterized in mammals: extracellular signal-regulated kinases (ERKs) 1 and 2 (namely ERK1/2), c-Jun amino-terminal kinases (JNKs) 1, 2, and 3, p38 isoforms α, β, γ, and δ, ERKs 3 and 4, and ERK5 (reviewed by Chen et al., 2001; Kyriakis and Avruch, 2001; Roux and Blenis, 2004). ERK 1 and 2 have been described to, when activated, phosphorylate membrane proteins (CD120a, Syk, and calnexin), nuclear substrates and cytoskeletal proteins (neurofilaments and paxillin) (reviewed by Chen et al., 2001; Roux and Blenis, 2004). Cyclic tensile forces up-regulate bone marrow protein-2 (BMP-2) expression via ERK1/2 and p38 MAP kinase pathways, with COX and PGE2 implication, in human periodontal ligament cells (Suzuki et al., 2014) and promote the migration of periodontal cells via ERK signaling pathway activation (Pan et al., 2010). Moreover, multiple data indicates that mechanical forces modulate ERK activities in periodontal fibroblasts increasing type I collagen, ostepontin and MMP-1 production (Liedert et al., 2006; Jeon et al., 2009; Kook et al., 2009, 2011).

Also related to extracellular stimuli-mediated signaling, Rho is a family of serine/threonine kinases (Wennerberg et al., 2005; Bustelo et al., 2007) involved in cell recruitment-migration, proliferation and apoptosis (Ridley, 2001; Etienne-Manneville and Hall, 2002). It has been recently described that Rho is involved in experimental orthodontic tooth movement by increasing Rho-kinase (ROCK) activity on the tension side (Meng et al., 2015). This fits with the finding that ROCK1 acts as a suppressor of inflammatory cell migration by regulating PTEN phosphorylation (Vemula et al., 2010). In line with this, it has been reported that Rho-ROCK enhances the formation of actin stress fibers and focal adhesion in fibroblasts (Amano et al., 1997). More concretely, during experimental orthodontic movement, the tension areas showed increased expression of actin stress fibers as well as increased number of myofibroblasts in the periodontal area (Meng et al., 2010, 2007). These facts are related to focal adhesion phenomena related to dental movement.

Not related to integrin-mediated signaling, Toll-like receptors (TLRs) are transmembrane proteins playing a critical role in innate immune system. TLRs are made up of an extracellular and of a cytoplasmic domain, homologous to the cytoplasmic domain of the human IL-1 receptor (Medzhitov, 2001). TLRs can recognize different patterns, known as pathogen-associated molecular patterns (PAMP). These PAMPs include lipids, proteins, lipoproteins, nucleic acids, and lipopolysaccarides (LPS) (Medzhitov and Janeway, 1997; Yang et al., 1998). TLR4 is particularly interesting in oral tissues because it is highly expressed by periodontal fibroblasts and specifically recognizes *Porphyromonas gingivalis* LPS (Takeuchi and Akira, 2001). The activation of TLR4 promotes pro-inflammatory signaling processes leading to periodontal alterations, osteoclast activation-recruitment and cytokine expression (Kikkert et al., 2007; Gelani et al., 2009; Nussbaum et al., 2009). TLR4 has been recently associated with mechanical forces on fibroblasts: its activation increased the expression of MMP-1, 3, and 10, increased phosphorylation of p38, JNK, and NF-κB, strongly suggesting that TLR4 may play an important role during orthodontic treatment (Lisboa et al., 2013). Hyaluronic acid (HA) is a classic and abundant component of connective tissue also present in the PDL. HA is an endogenous ligand for TLR4 that promotes protective responses in skin and lung injury models (Jiang et al., 2005; Taylor et al., 2007). Although the anti-inflammatory properties of HA and its mechanisms are partially unknown, direct interactions with inflammatory cells and the physical properties of the molecule, seem to be implicated. It is shown that HA reduces TNF-α and IFN-γ production and induces NF-κB activation in macrophages (Noble et al., 1996; Wang et al., 2006). As an example, this TLR4-HA interaction seems to be related to Cox-2 and PGE_2_ production to protect the colon mucosa from injury (Chen et al., 2011). More research is needed to explain the concrete role and mechanism of the HA-TLR4 interaction that could make it be of interest for orthodontic and periodontal clinical care.

**Figure 1:**
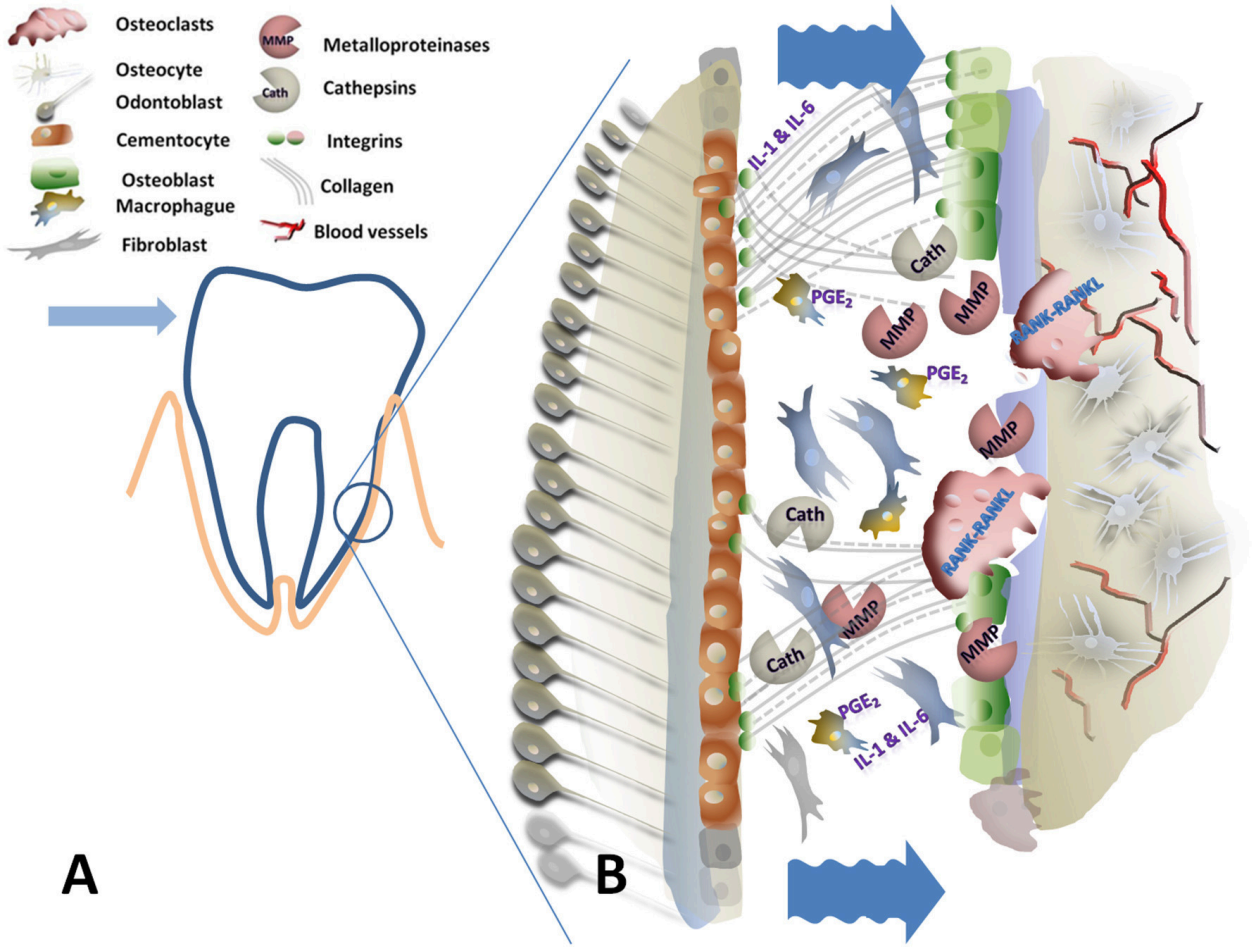
**Graphical scheme of the periodontal ligament and alveolar bone under compressive forces. (A)** Is the schematic representation of a tooth in the socket where the arrowhead indicates the sense of the applied force and the encircled area represents the compressive side. **(B)** Detailed view of the compressive side: Wrinkled arrows indicate the reduction on the periodontal gap due to compression. This mechanical signal affects cells and extracellular matrix components promoting extracellular release of matrix degrading enzymes as Metalloproteinases (MMP) and Cathepsins (Cath), macrophage activation (IL1, 6, and PGE2) and RANK-RANKL osteoclast activation. This results on bone resorption with periodontal destruction-reconstruction in the new dental position.

**Figure 2:**
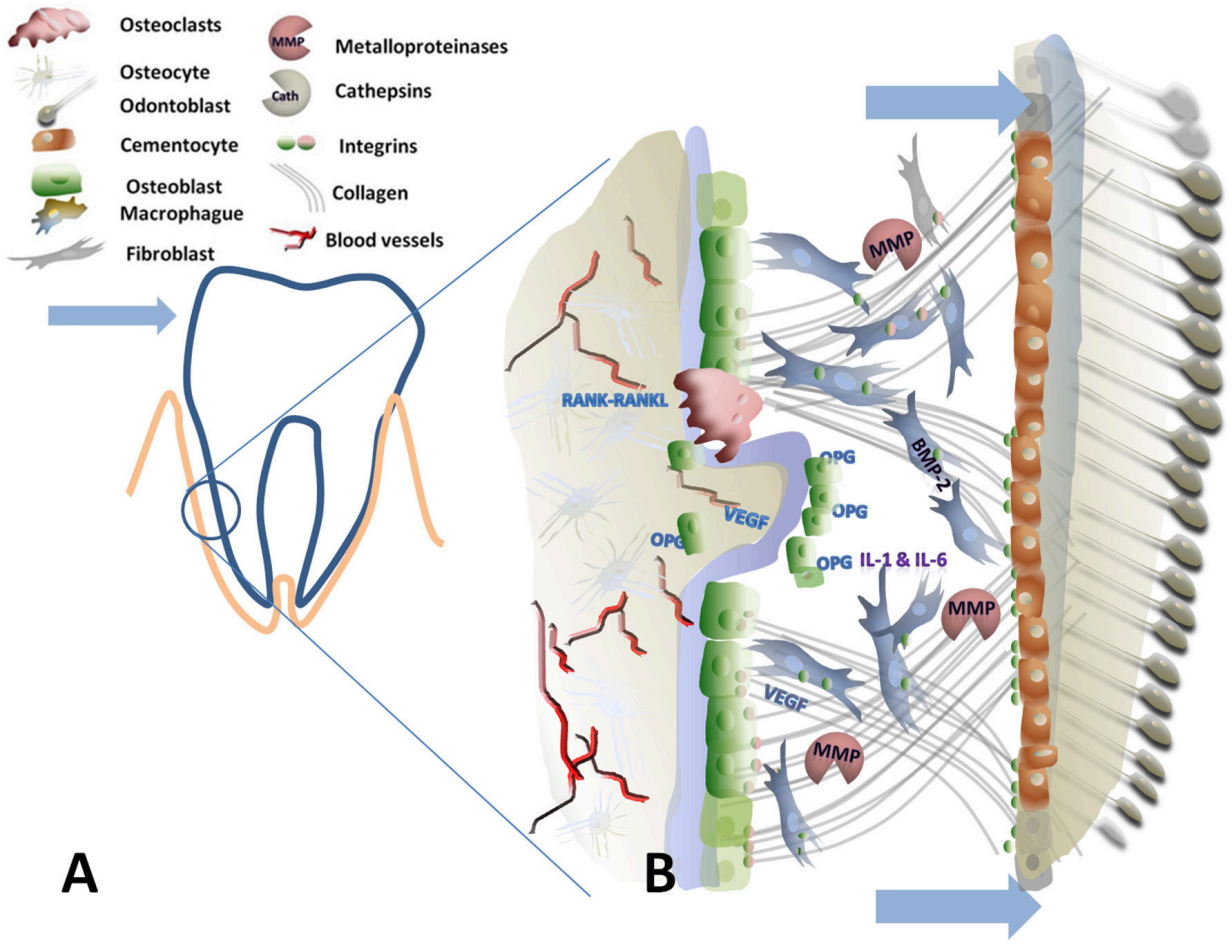
**Graphical scheme of the periodontal ligament and alveolar bone under tensile forces. (A)** Is the schematic representation of a tooth in the socket where the arrowhead indicate the applied force. The encircled area represents the tensile side. **(B)** Detailed view of the tensile side: Arrows indicate the increase on the periodontal gap due to tensile force. Tensile forces are transmitted via collagen-coupled Integrins to different cell types promoting new bone generation. Bone marrow protein 2 (BMP-2), osteoprotegerin (OPG), vascular endothelial growth factor (VEGF) are necessary for new bone formation.

A graphic summary of the periodontal area with the extracellular processes is detailed in Figures 1, 2.

## EtOH modulates extracellular proteins and promotes intracellular changes

### EtOH modifies extracellular protein activities and bone dynamics

It is well documented how EtOH affects osteoclastic/osteoblastic dynamics producing osteopenia and osteoporosis (Manolagas, 2000; Turner, 2000). Although the mechanisms are not fully understood, EtOH may promote bone loss inhibiting osteoblastogenesis (Friday and Howard, 1991) by altering bone remodeling-related genes (Chakkalakal, 2005; Callaci et al., 2009). It has also been shown an inverse correlation between EtOH intake and bone mineral density in both pre- and post-menopausal women (Turner and Sibonga, 2001). IL-6 seems to be responsible, at least in part, for this EtOH-induced bone loss (Dai et al., 2000). Interestingly enough, IL-6 is also increased during orthodontic movement (Grieve et al., 1994; Uematsu et al., 1996) and therefore it seems plausible that EtOH consumption during orthodontic treatment would affect the outcome of the intervention via IL-6. More research is needed to analyze the IL-6 levels and bone remodeling under these circumstances (EtOH+ orthodontic forces).

Some reports indicate that EtOH exposure preferentially alters the periodontal area, developing periodontitis by increasing the loss of attachment through recession of gingival margins (Khocht et al., 2003) or by altering the oral mucosa (Harris et al., 1996, 2004). Regarding the influence of EtOH and other drugs on tooth decay, some studies focus the attention on the EtOH-induced oral micro-flora alterations due to EtOH-acetaldehyde metabolism, leading to the progression of dental caries (Dasanayake et al., 2010; Rooban et al., 2011), and little is known about the role of EtOH on orthodontic movement.

Estrogens can protect from bone resorption (Kousteni et al., 2001; Chen et al., 2005) and this inhibitory effect seems to be related to the RANKL-RANK-OPG system (Syed and Khosla, 2005). In fact, estrogens can suppress RANKL expression in osteoblasts (Bord et al., 2003). Chen et al. (2006, 2008) found that the protective effects of estradiol on EtOH-induced bone loss was related to the inhibition of ROS production in osteoblasts. Additionally, NADPH oxidase (NOx) and estradiol would play a critical role on EtOH-induced bone loss via the ERK/STAT3/RANKL pathway.

It has been demonstrated that chronic EtOH consumption promotes bone loss, increases PGE2 expression and other inflammatory markers in rats (Dantas et al., 2012; Surkin et al., 2014). All these markers are related to periodontal disease, so the hypothesis of EtOH-induced oxidative burden as a modulator of the extracellular environment during tooth movement is supported.

Some reports indicate that MMP-1, Cathepsins K, B, and L are increased in the compression side during tooth movement (Domon et al., 1999; Sugiyama et al., 2003; Maeda et al., 2007). EtOH-induced osteoclastogenesis increases RANK and Cathepsin K activities (Domon et al., 1999). However, it has been shown that EtOH reduces proteolytic activity in hepatic Cathepsins B and L (Kharbanda et al., 1995, 1996). EtOH and tooth movement may act in the same way by increasing bone resorption and PDL remodeling in the compression side despite this tissue difference. Future studies should be addressed to know whether this fact is synergistic or accumulative leading to excessive bone resorption and eventually to root resorption.

### EtOH promotes intracellular responses

EtOH affects intestinal epithelial tight junction integrity via Ca^++^-mediated Rho/ROCK activation (Elamin et al., 2014). It has been described that EtOH exposure disorganizes actin-cytoskeleton in astrocytes and this process is mediated by RhoA signaling pathway (Guasch et al., 2003). Although nothing is known about the effect of EtOH on the cytoskeletal periodontal fibroblasts and osteoblasts, some evidence indicates that both EtOH and periodontal movement act in the same Rho-ROCK pathway. It seems reasonable that EtOH exposure during orthodontic movement may alter the cytoskeletal organization affecting orthodontic outcome.

ROS is a relevant extracellular ERK-triggering stimulus that up-regulates ERK-dependent genes such as RANKL (Torres, 2003). Supporting this fact, it was found that the administration of antioxidants such as N-Acetyl cysteine, estradiol or vitamin C, suppress RANKL mRNA expression and induces PDL progenitor cell differentiation via ERK activation pathway (Chen et al., 2008; Yan et al., 2013). In this regard, it is well documented that EtOH metabolism results in ROS production and subsequently leads to cell damage and eventually death (Johnsen-Soriano et al., 2007; Flores-Bellver et al., 2014). Even more, EtOH-induced oxidative stress seems to be crucial for these negative effects on cells, since the administration of antioxidants restores the oxidative misbalance and prevents the negative effects on cells (Herrera et al., 2003; Koch et al., 2004; Crews et al., 2007). ROS and EtOH activate MMP- 1,-2, and -9 via protein tyrosine kinase signaling, leading to basal membrane disruption (Haorah et al., 2007, 2008).

**Figure 3:**
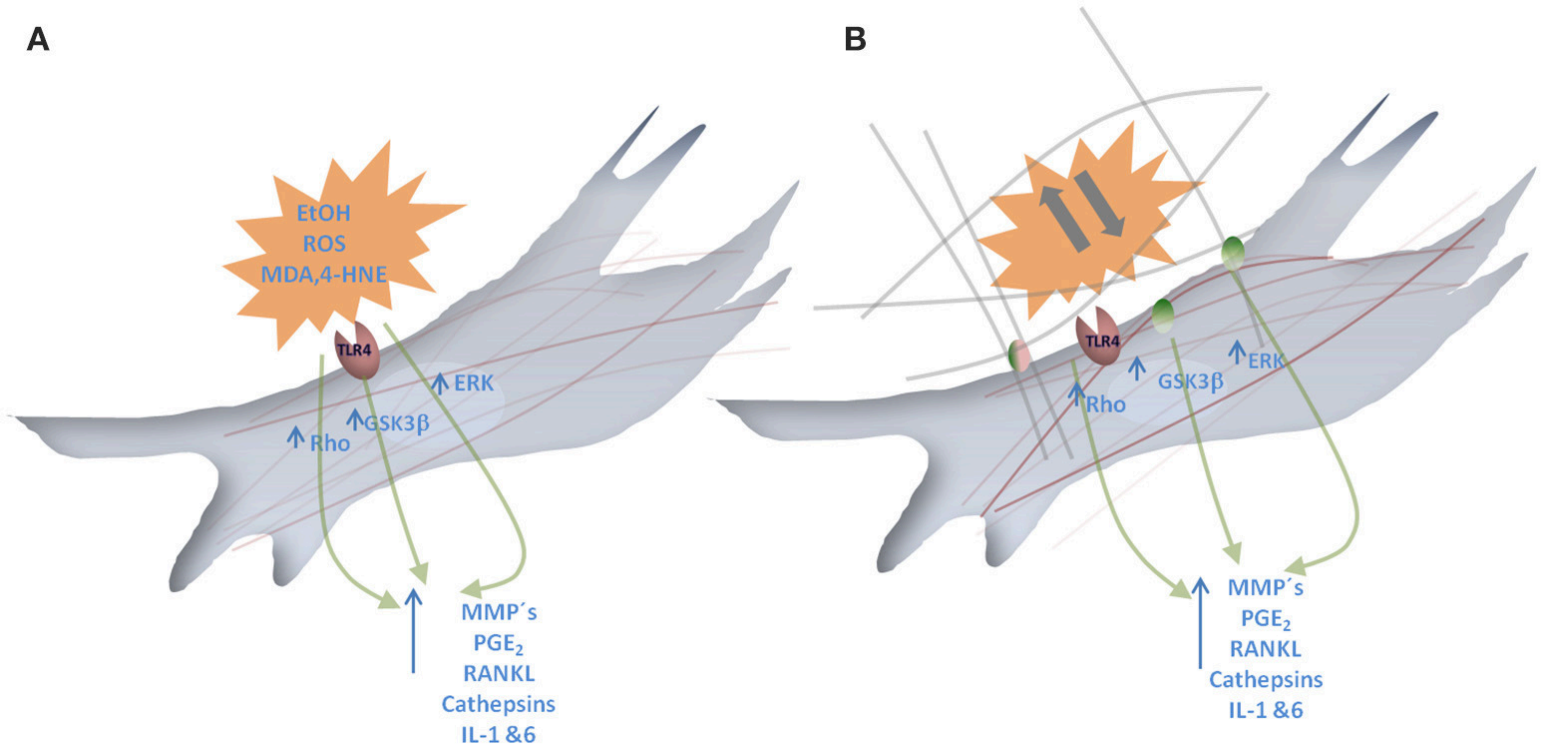
**Graphical scheme of the molecular responses promoted by EtOH exposure and mechanical forces. (A)** Represents a fibroblast where EtOH and EtOH-derived reactive oxygen species (ROS), lipid peroxidation products such as 4-hydroxynonenal (4-HNE) and Malondialdehyde (MDA) interact with Toll like receptor4 (TLR4). This interaction results in Rho, GSKβ and ERK pathway activation that leads to MMP/Cath, PGE_2_, RANKL and IL-1 and 6 release. **(B)** Representation of a fibroblast where mechanical forces affecting extracellular matrix can interact with integrins. This interaction results in Rho, GSKβ, and ERK pathway activation that leads to MMP/Cath, PGE_2_, RANKL and IL-1 and 6 release.

It is well documented that EtOH promotes inflammatory responses via TLR4 in different tissues, e.g., brain, lung and liver (Vaneker et al., 2008; Fernandez-Lizarbe et al., 2013; Zmijewski et al., 2014; Pascual et al., 2015). So, after considering the aforementioned data on TLR4, it seems plausible that EtOH exposure could be closely related to periodontal stability (see Figure 3) and therefore it becomes an important factor on clinical practice.

Glycogen synthase kinase 3 β (GSK3β) regulates the production of cytokines after TLR4 stimulation (Martin et al., 2005). TLR4-GSK3β route activation has been closely related to periodontal alterations induced by *P. gingivalis* and other pathogens (Wang et al., 2011). In this sense, and fitting with this, it has been shown how alcoholic fatty liver pathogenesis implicates GSK3β route activation (Zeng et al., 2014) and it has been demonstrated that GSK3β inhibition suppresses bacterial-induced periodontal bone loss (Adamowicz et al., 2012), supporting the idea that TLR4-GSK3β pathway could be particularly affected in alcohol-users. It is well documented that EtOH promotes inflammatory responses via TLR4 in different tissues, e.g., brain, lung and liver (Vaneker et al., 2008; Fernandez-Lizarbe et al., 2013; Zmijewski et al., 2014; Pascual et al., 2015). Considering the aforementioned data on TLR4, it seems plausible that EtOH exposure could be closely related to periodontal stability (see Figure 3) and therefore it becomes an important factor on clinical practice.

## Final discussion

Despite the fact that several studies indicate the potential deleterious effect of EtOH on periodontum and alveolar bone, there is only one report on the effects of ethanol during orthodontic movement. In this work, Araujo and collaborators describe less bone resorption at the end of tooth movement suggesting a delay of tooth movement in a rat model of binge drinking (Araujo et al., 2014). Obviously, this model simulates a drinking pattern where high EtOH concentrations are acutely consumed, which is different from the chronic pattern, where high EtOH levels are daily maintained for several weeks. According to the typically long lasting orthodontic treatments, mild and chronic EtOH exposure could interfere with this chronic orthodontic treatment by modifying the aforementioned proteins and genes leading to orthodontic failure or undesired outcome.

Systemic diseases are of relevance in oral medicine and dentistry. *Diabetes mellitus* (DM) is considered a common systemic disease with oral manifestations and profuse literature deals with the considerations of orthodontic treatment on diabetic patients (Burden et al., 2001; Vernillo, 2001; Bensch et al., 2003; McKenna, 2006). Experimental data widely show that DM promotes molecular and structural changes in the periodontal area after orthodontic treatment including MMP's or bone alterations (Feng et al., 2007; Abbassy et al., 2010; Braga et al., 2011; Villarino et al., 2011; Zhang et al., 2011). There are some similarities between alcohol exposure and DM in terms of molecular signaling and gene expression (Barcia et al., 2015). Additionally, experimental and clinical studies strongly indicate a close relationship between alcohol intake and risk of diabetes development (Cullmann et al., 2012; Kim et al., 2013). In consonance with this issue, orthodontic movement promotes intra and extracellular alterations, finally affecting periodontum and alveolar bone. Since the influence of DM in orthodontic treatment outcome seems clear, it seems appropriate to further investigate the effects of chronic EtOH exposure on orthodontic treatment.

As a hypothetical model, EtOH exposure during orthodontic movement may interfere with osteogenesis at the tension side, accepting that EtOH produces osteoblastogenesis inhibition (Friday and Howard, 1991). IL-6/ROS and PGE2 mediated bone loss is induced by EtOH (Dai et al., 2000; Chen et al., 2006) and it also increases RANKL (Chen et al., 2008). Additionally, as mentioned above, TLR4 over-activation (EtOH+ tensile strain) may lead to GSKβ activation, negatively affecting the periodontum (Kikkert et al., 2007; Gelani et al., 2009; Nussbaum et al., 2009). On the compression side, where bone destruction and reorganization takes place, probably bone resorption would be increased during EtOH metabolism leading to a rapid but unstable teeth position.

In view of the close similarities found between EtOH- and mechanical strain-induced responses on periodontal tissues, the aim of this review is to spark attention on the potential effect of EtOH consumption during orthodontic or periodontal treatment as a factor that needs to be considered in clinical practice. Further research is necessary to fully and experimentally support the actual indications suggesting that alcoholic beverages consumption should be discouraged during orthodontic treatment in adults.

## Author contributions

JB proposed the subject of the revision and distributed the tasks; supervised each of the topics that were revised. SP, VA, and LP revised the existing literature on tooth movement regulation and effects of alcohol on oral diseases. AU and GP supervised references content and the different perspectives studied. VV and VA supervised manuscript writing. FR supervised the whole manuscript and its final version.

### Conflict of interest statement

The authors declare that the research was conducted in the absence of any commercial or financial relationships that could be construed as a potential conflict of interest.
